# A Comparison of Adult Mosquito Trapping Methods to Assess Potential West Nile Virus Mosquito Vectors in Greece during the Onset of the 2018 Transmission Season

**DOI:** 10.3390/insects11060329

**Published:** 2020-05-27

**Authors:** Marina Bisia, Claire L. Jeffries, Ioanna Lytra, Antonios Michaelakis, Thomas Walker

**Affiliations:** 1 Department of Disease Control, Faculty of Infectious and Tropical Diseases, London School of Hygiene and Tropical Medicine, London WC1E 7HT, UK; mbisia@outlook.com (M.B.); claire.jeffries@lshtm.ac.uk (C.L.J.); 2 The Royal Veterinary College, London NW1 0TU, UK; 3 Department of Entomology and Agricultural Zoology, Benaki Phytopathological Institute, 14561 Athens, Greece; i.lytra@bpi.gr (I.L.); a.michaelakis@bpi.gr (A.M.)

**Keywords:** mosquitoes, West Nile virus, *Culex pipiens* complex, molecular xenomonitoring

## Abstract

West Nile virus (WNV) threatens the health of humans and equines worldwide. *Culex* (*Cx.*) *pipiens* complex mosquitoes are major vectors but numerous other species have been implicated. Due to variations in blood-feeding behaviour, *Cx. pipiens* biotypes and hybrids influence transmission, from enzootic cycles (between mosquitoes and birds), to spill-over transmission to humans and equines. In this study, mosquitoes were collected in May–June 2018 during the early period of the transmission season from two regional units of Greece, where WNV cases had been reported in the previous four years (Palaio Faliro and Argolida). A total of 1062 mosquitoes were collected with Biogents Sentinel 2 traps collecting both a greater number of all mosquito species and the *Cx. pipiens* complex than CDC miniature light traps or Heavy Duty EVS traps. Molecular identification confirmed additional species including *Aedes albopictus.* The proportion of *Cx. pipiens* biotypes in Palaio Faliro was 54.5% *pipiens*, 20.0% *molestus* and 25.5% hybrids. In Argolida, the collection comprised 68.1% *pipiens* biotype, 8.3% *molestus* biotype and 23.6% hybrids. Screening resulted in WNV detection in three females of the *pipiens* biotype and in one hybrid. As hybrids play a role in spill-over transmission, these findings highlight the importance of entomological surveillance programs incorporating molecular xenomonitoring as an early warning before human cases at the onset of the transmission season.

## 1. Introduction

West Nile virus (WNV) is an arbovirus belonging to the Japanese encephalitis serocomplex within the *Flavivirus* genus (*Flaviviridae* family) and is the most widespread flavivirus, with circulation worldwide, including the USA and Europe [1,2,3,4]. Natural transmission of WNV mainly occurs in enzootic cycles between birds and competent ornithophilic mosquito vectors, with avian species being the principal maintenance and amplifying hosts of WNV as many species develop sufficient viremia for onward transmission [5,6,7]. Enzootic transmission can continue onward where infected mosquitoes are present in a specific area under suitable environmental conditions [8]. Additionally, spill-over transmission can occur when competent vectors feed on humans or horses. During natural transmission both humans and horses are considered dead-end hosts since they cannot sustain sufficient viraemia for further vector-borne transmission [9]. However, infection in humans does pose a transmission risk due to the possibility of iatrogenic transmission through blood and tissue donations, in addition to the possibility of intrauterine transmission or WNV being passed on through breast milk [4]. Blood and tissue donor screening is essential in areas where WNV is endemic [10,11]. Although currently no human vaccination is available, vaccination of horses has been shown to reduce clinical disease within this species [12,13].

WNV was first isolated in 1937 from a woman with febrile illness in the West Nile district of Uganda [14]. WNV has caused numerous annual outbreaks in North America and Europe leading to major concern for human and animal health [3,15]. In North America, the majority of arboviral encephalitis cases are attributable to WNV [16]. Although ~80% of human WNV infections are asymptomatic, the broad clinical spectrum can result ranging from a mild flu-like illness in ~20% of infected individuals (West Nile fever) to severe neurological disease through infection of the central nervous system (<1% of infected individuals) that can lead to death from meningitis, encephalitis and acute flaccid paralysis [17,18]. Therefore, a high proportion of asymptomatic infections highlights that the number of human cases demonstrating overt disease, or discovered through laboratory testing, are likely just the ‘tip of the iceberg’ of the actual number of viral infections occurring within a population [19]. Furthermore, these spill-over infections in humans are likely to be far less frequent compared to the amount of enzootic transmission occurring between mosquitoes and avian species [20].

The introduction and spread of WNV in Europe is thought to have been driven by migratory birds [21,22,23,24]. WNV resulted in sporadic human cases from the mid-1990s [25] but was considered to be an increasing public health concern with the first large outbreak in Europe occurring in Romania in 1996 with 393 hospitalised cases and 17 deaths [26]. From 2010, the European Centre for Disease Control (ECDC) have monitored WNV cases in the European Union and neighbouring countries and publishes weekly epidemiological reports [27]. In Greece, WNV was first detected in the summer of 2010 in the central Macedonia Region near the city of Thessaloniki, in the northern part of the country [28,29]. This outbreak included 262 probable and confirmed cases of WNV infection of which 197 were neuroinvasive cases and 35 deaths [30]. In 2011 WNV was found in both humans and horses; detected from clinical and laboratory surveillance techniques [31]. In the following years, cases of WNV in humans and animals were reported in central Greece and in the Attica Region but there were no reported cases in 2015 or 2016 [31]. In 2017, WNV re-emerged in southern Greece and in 2018 there were 311 laboratory confirmed human cases, resulting in 47 deaths, showing a marked increase over 2017, with only 48 confirmed cases and 5 deaths [27,31]. Historical data of human cases with neurological disease in Greece from 2010 until present show that cases increase in August (the peak month in the transmission season) and prior to 2018, the largest case numbers per month were reported in August 2010 [30,31,32]. There have been over 60 species of mosquitoes in the USA implicated as potential WNV vector species [4]. Seven of these species occur in Europe and have been tested for WNV susceptibility using laboratory vector competence experiments including members of the *Cx. pipiens* complex, *Ae. albopictus* and *Ae.* (*Ochlerotatus*) *caspius* [33]. WNV transmission rates of European *Cx. pipiens* complex mosquitoes ranged between 0–60%, 40–55% for *Cx. modestus*, 0–40% for *Ae. albopictus* and 0–1% for *Ae. caspius* (reviewed in [33]). Temperature has been shown experimentally to increase WNV transmission rates of the *pipiens* and *molestus* biotypes, in addition to the sibling species *Cx. torrentium* [34]. According to the ECDC, the most important vector species for the transmission of WNV to humans present in Europe are *Cx. pipiens* s.l. and *Cx. modestus* [35]. This year, horizontal and vertical transmission of WNV by *Ae. vexans* was also confirmed [36].

The *Culex pipiens* complex consists of morphologically indistinguishable species including *Cx. pipiens*, *Cx. quinquefasciatus*, *Cx. pallens* and *Cx. australicus* that have varied behaviour, physiology and host preference [37,38]. Within Europe, *Cx. torrentium* is also often morphologically indistinguishable from species in the *Cx. pipiens* complex [39]. *Culex pipiens* has two behaviourally different biotypes, *pipiens* and *molestus*, which can form hybrids, and their feeding behaviours can influence their role in local transmission of WNV. The two biotypes are morphologically indistinguishable but have genetic, biological and behavioural differences. The *pipiens* biotype is anautogenous, so females need to consume a blood meal to lay eggs [40]. Furthermore, the *pipiens* biotype requires a large space to swarm for mating and are found above ground undergoing diapause. Although the *pipiens* biotype is considered an important species for the enzootic WNV transmission cycle given its preference to feed on birds [41,42], other studies have shown diverse host-feeding patterns including the occurrence of human derived blood meals in addition to mixed avian-human blood meals [43]. In contrast, the *molestus* biotype is autogenous and can lay eggs without a blood meal. Mating can happen in confined spaces, while they live underground, do not undergo diapause and are more anthropophilic, preferentially feeding on humans. The *molestus* biotype and hybrids are implicated in the spill-over transmission of WNV from avian hosts to humans due to the opportunistic feeding behaviour of the *molestus* biotype [42,43]. In Greece, hybrids have previously been detected [44] in addition hybrids of *pipiens* and *quinquefasciatus* on the Kos Island [45].

In order to better understand the complexity of WNV transmission, entomological surveys for arboviral surveillance can be undertaken to determine both the presence of potential mosquito vectors and provide evidence for WNV circulation through virus detection in field-caught mosquitoes (molecular xenomonitoring). Entomological surveillance could provide an important role in the monitoring and prevention of major outbreaks. Here we report the results of an entomological survey undertaken at pre-disease stage (no autochthonous cases detected in animals or humans) in two Regional Units (RUs) of Greece (Palaio Faliro in the Attica region and Argolida in the Peloponnese region) where WNV outbreaks have previously been recorded. We compared the mosquito species abundance and diversity using Biogent sentinel 2 (BG) traps, Heavy duty Encephalitis Vector Survey (EVS) traps and Centre for Disease Control miniature light (CDC) traps. We determined the prevalence of the *Cx. pipiens* biotypes (*pipiens*, *molestus* and hybrids) in each sampling location and female mosquitoes were screened for the presence of WNV to determine whether there was any evidence of virus circulation in the two RUs.

## 2. Materials and Methods

### 2.1. Mosquito Collections

**Figure 1:**
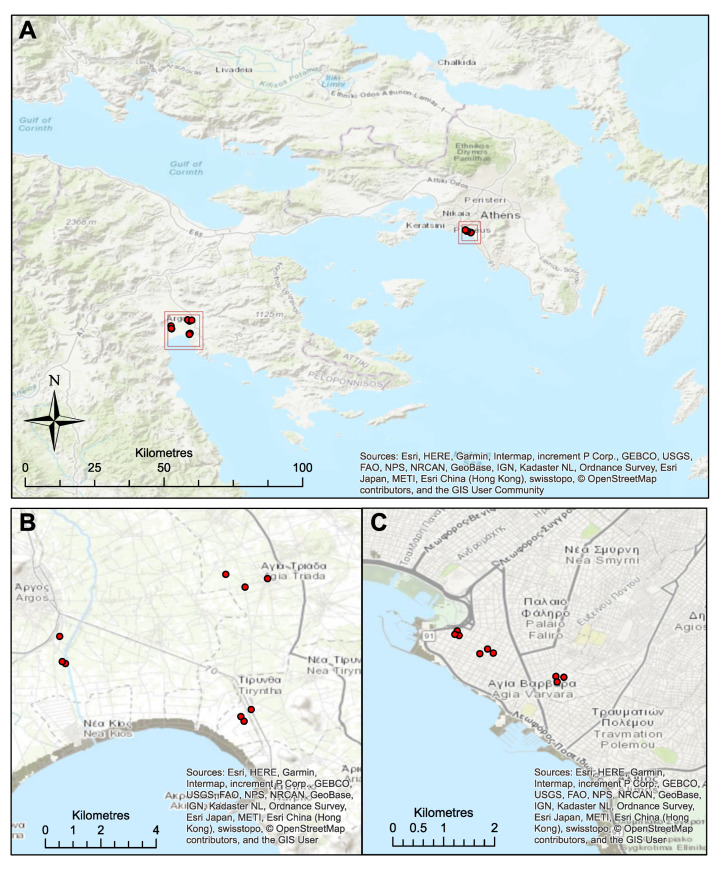
(**A**) Locations of collection sites within the Regional Unit of Argolida in the Peloponnese region and within the Regional Unit of Palaio Faliro in Attica the region; (**B**) sampling locations with the trapping sites within Argolida and (**C**) Palaio Faliro. Maps constructed in ArcMap 10.5 (Esri, ArcGIS), using World Topographic Basemap and GPS coordinates from trapping sites.

The study was carried out in two Regional Units (RUs) within the Attica and Peloponnese regions of Greece, with three sampling locations selected from within each RU, and three trapping sites within each sampling location (Figure 1, Table S1). Locations for trapping in the RU of Palaio Faliro were classified as urban, whereas those in the RU of Argolida were rural. In each sampling location, three different traps (trapping sites) were operating for 24 h, three times per week on consecutive days. Trapping occurred over a six-week period (May–June 2018) during the start of the WNV transmission season (based on previous historical data obtained from ECDC [27]). Traps were setup on fully charged 12 V batteries at 14:00 and run until 09:00 the following day to provide overnight collections. A 3 × 3 design was applied at each site to minimize site and environmental confounding factors and traps were placed more than 100 m from each other and rotated every 24 h between selected positions so that each trap had been used in every site. Three different trap types were used in each site to maximise the diversity of species collected; BG2 traps (Biogents, Regensburg, Germany), Heavy Duty Encephalitis Vector Survey trap (EVS trap) (BioQuip Products, Rancho Dominguez, California, USA) and CDC traps (John W. Hock, Gainesville, Florida, USA). Dry ice was used as an attractant in all traps with approximately 2 kg/rap per 24 h. All traps were run for six consecutive weeks (four in Palaio Faliro followed by two in Argolida). Mosquitoes were collected every 24 h, killed on dry ice and stored at −80 °C. Morphological keys were used to identify individuals to species or species complex level [46] 8–10 days after collection using a large (100 mm × 20 mm) polysterene petri dish sat on a 1:1 mixture of ice and acetone. Female mosquitoes were classified as unfed (no evidence of blood in their abdomen), blood-fed or gravid. Individual mosquitoes were then placed in RNAlater (Invitrogen) to preserve RNA for downstream molecular analysis.

### 2.2. DNA/RNA Extraction and cDNA Synthesis

DNA was extracted from individual male mosquitoes using QIAGEN DNeasy Blood and Tissue Kits (Qiagen, Manchester, UK) according to manufacturer’s instructions as WNV virus screening was not undertaken on males. DNA extracts were eluted in a final volume of 100 μL and stored at −20 °C. RNA was extracted from individual female mosquitoes using Roche High Pure RNA Isolation Kits (Roche Diagnostics, Mannheim, Germany) and QIAGEN RNeasy 96 kits (Qiagen, Manchester, UK) according to manufacturer’s instructions. RNA extracts were eluted in a final volume of 45 μL and stored at −80 °C. RNA was reverse transcribed into complementary DNA (cDNA) using an Applied Biosystems High Capacity cDNA Reverse Transcription kit (USA). A final volume of 20 µL contained 10 µL RNA, 2 µL 10x RT buffer, 0.8 µL 25x dNTPs (100 mM), 2 µL 10x random primers, 1µL reverse transcriptase and 4.2 µL nuclease-free water. Reverse transcription was undertaken in a Bio-Rad T100 Thermal Cycler as follows: 25 °C for 10 min, 37 °C for 120 min and 85 °C for 5 min, with the cDNA stored at −20 °C.

### 2.3. Molecular Identification of Species

Species previously shown to be potential WNV vectors were first identified through Sanger sequencing of conserved cytochrome c oxidase 1 (*CO1)* gene fragments [47,48,49]. As in previous studies looking at diverse mosquito species, amplifying and sequencing different *CO1* regions was required for confirmation of different species. Specimens identified as within the *Cx. pipiens* complex were further identified to species level using a combination of multiplex species-specific PCR assays given the inability to discriminate species through sequencing *CO1* regions [37,50]. PCR products were separated and visualized using 2% E-gel EX agarose gels (Invitrogen) with SYBR safe and an Invitrogen E-gel iBase Real-Time Transilluminator. PCR products were submitted to Source BioScience (Source BioScience Plc, Nottingham, UK) for PCR reaction clean-up, followed by Sanger sequencing to generate both forward and reverse reads. Sequencing analysis was carried out in MEGA7 [51] as follows. Both chromatograms (forward and reverse traces) from each sample was manually checked, analysed and edited as required, followed by alignment by ClustalW and checking to produce consensus sequences. Consensus sequences were used to perform nucleotide BLAST (NCBI) database queries and sequences were compared to those available from GenBank (NCBI). Representative full consensus sequences for *CO1* gene fragments were submitted to GenBank and assigned accession numbers MN005042-MN005056.

### 2.4. WNV Screening

Screening for WNV detection was undertaken on cDNA resulting from individual female mosquito RNA extracts using a WNV-specific real-time PCR assay [52]. Reactions were prepared using 5 µL of Qiagen QuantiTect SYBR^®^ Green Master mix, a final concentration of 1 µM of each primer, 1 µL of PCR grade water and 2 µL template cDNA, to a final reaction volume of 10 µL. Prepared reactions were run on a Roche LightCycler^®^ 96 System and PCR cycling conditions were as follows: 95 °C for 10 min followed by 45 cycles of 95 °C for 10 s, 60 °C for 10 s, 72 °C for 20 s. PCR products were also separated and visualised using 2% E-Gel EX agarose gels (Invitrogen) with SYBR safe and an Invitrogen E-Gel iBase Real-Time Transilluminator to confirm successful amplification of the 144 base pair target fragment.

### 2.5. WNV Case Mapping

Human WNV reported cases were mapped for the year of collection to provide context to the entomological survey. Maps were constructed in ArcMap 10.5 (ArcGIS, Esri, Redlands, USA) using Global Administrative layers for Greece (level 3), downloaded from www.gadm.org (Version 3.6) and anonymized ECDC WNV case report data from “Transmission of West Nile virus, June to December 2018—Table of cases, 2018 transmission season” downloaded from www.ecdc.europa.eu. The EU NUTS (Nomenclature of territorial units for statistics) level 3 regions as listed in the ECDC data sheet were matched to the Global Administrative layer level 3 (municipalities) during map construction, with each of the GADM (Database of Global Administrative Areas) level 3 municipalities matched to the corresponding NUTS level 3 region and assigned the same reported case data. The data from the ECDC surveillance Atlas was collected for each week of the transmission season, for human and equine cases, and then combined for each region, to generate monthly maps of human case reports, and cumulative total maps for human and equine cases.

### 2.6. Statistical Analysis

Non-parametric Mann Whitney U tests were performed in Microsoft Excel (version 16.21.1) to compare the number of *Cx. pipiens* complex mosquitoes for each trap type in a given sampling location.

## 3. Results

### 3.1. Mosquito Species Abundance and Diversity

A total of 1062 mosquitoes comprising 840 unfed females, 28 blood-fed females, 9 gravid females and 185 males were captured (Table 1). Species belonging to the *Cx. pipiens* complex were the most abundant, comprising 62.5% (*n* = 664) of the total collection across both RUs. Additional species collected included *Culiseta (Cs.) longiareolata* (16.1%, *n* = 171), *Ae. caspius* (11.0%, *n* = 117), *Ae. albopictus* (7.4%, *n* = 79) and species belonging to the *Anopheles (An.) maculipennis* complex (1.8%, *n* = 19). The remaining 1.1% (*n* = 12) of mosquitoes were not possible to morphologically identify using keys due to damage during trapping. Individuals of the *Cx. pipiens* complex and *Cs. longiareolata* specimens were collected from all sites within both regions. In the RU of Palaio Faliro, Attica region, *Ae. albopictus* specimens were collected in all three sites and single individuals were also collected in Agia Triada and Dalamanara within the RU of Argolida. In contrast, *Ae. caspius* and *An. maculipennis* complex individuals were collected in all three sites within the RU of Argolida, but not from sites within the RU of Palaio Faliro.

### 3.2. Species Trap Comparison

In both RUs, BG traps collected both more overall mosquitoes of all species, and a greater number of specimens from the *Cx. pipiens* complex, than CDC traps and EVS traps (Table 2). As the data was not normally distributed, non-parametric Mann–Whitney tests were used to determine any significant differences in the number of *Cx. pipiens* complex mosquitoes collected using different trap types (Table 2). In the RU of Palaio Faliro, BG traps collected more *Cx. pipiens* complex mosquitoes (*n* = 101) than CDC (*n* = 46) and EVS (*n* = 41) traps although the comparison between BG and CDC traps was not statistically significant (Mann-Whitney U = 258.0, *p* = 0.07). In the RU of Argolida BG traps collected significantly more *Cx. pipiens* complex (*n* = 214) than CDC (*n* = 69) and EVS (*n* = 50) traps (Mann–Whitney U = 40, *p* = 0.02; U = 32, *p* = 0.01, respectively).

### 3.3. Molecular Identification of Species

**Figure 2:**
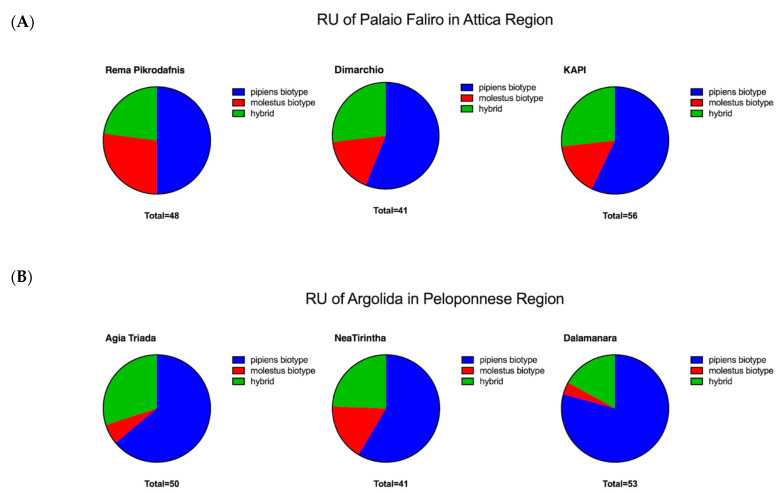
Prevalence rates of *Cx. pipiens* biotypes. Multiplex species-specific PCR assays were undertaken on *Cx. pipiens* complex individuals from three sampling locations in (**A**) the Regional Unit (RU) of Palaio Faliro in the Attica region and (**B**) the RU of Argolida in the Peloponnese region of Greece during May–June 2018.

Sanger sequencing of *CO1* gene fragments [47,48,49] was undertaken to confirm morphological identification of species and to also determine the species of morphologically unidentified specimens that had been damaged during trapping. Representative *CO1* gene fragment sequences from individuals of the *Cx. pipiens* complex from all six collection sites across both RUs did not produce sufficient sequence variation to determine biotypes (Table 3). Sequencing an additional *CO1* fragment [48] successfully confirmed the identification of *Cs. longiareolata* (*n* = 3) and *Ae. albopictus* (*n* = 3). Sequencing of an alternative *CO1* fragment [47] was required to successfully confirm *Ae. caspius* (*n* = 3) due to unsuccessful amplification of other *CO1* fragments. Speciation of a larger number of *Cx. pipiens* complex individuals (~40% of individuals from each location using different trapping types) was undertaken using multiplex species-specific assays [37,50] to determine the *pipiens*, *molestus* and hybrid biotypes. Multiplex species-specific assays revealed the presence of both biotypes of *Cx. pipiens* (*pipiens* type and *molestus* type) in addition to hybrids (Figure 2). In the RU of Palaio Faliro overall 54.5% (*n* = 79) were confirmed as the *pipiens* type, 20.0% (*n* = 29) as the *molestus* type and 25.5% (*n* = 37) as hybrids. In the RU of Argolida, 68.1% (*n* = 98) were *pipiens* type, 8.3% (*n* = 12) *molestus* type and 23.6% (*n* = 34) hybrids.

### 3.4. WNV Infection Rates in Field Mosquitoes

A total of 630 individual mosquitoes (229 from RU of Palaio Faliro and 401 from RU of Argolida) were screened for the presence of WNV cDNA. This included individual mosquitoes from the *Cx. pipiens* complex (*n* = 458), *Ae. caspius* (*n* = 114), *Ae. albopictus* (*n* = 31), *An. maculipennis* complex (*n* = 15) and unidentified mosquitoes (*n* = 12). In total, four *Cx. pipiens* complex individuals were WNV positive with no evidence of infection in any of the other species/species complexes. Real-time PCR results were confirmed by running PCR products through gel electrophoresis to confirm the correct target 144 base pair PCR products (Open Science Framework: DOI 10.17605/OSF.IO/D76QF). These positive individuals were unfed females which were molecularly identified as three *pipiens* biotype and one hybrid biotype, all collected from Dalamanara within the RU of Argolida in the Peloponnese region. Two WNV-infected mosquitoes were found on consecutive days (30 May 2018, 31 May 2018) in two sites (termed private house 1 and private house 2) in Dalamanara.

### 3.5. WNV Reported Cases

**Figure 3:**
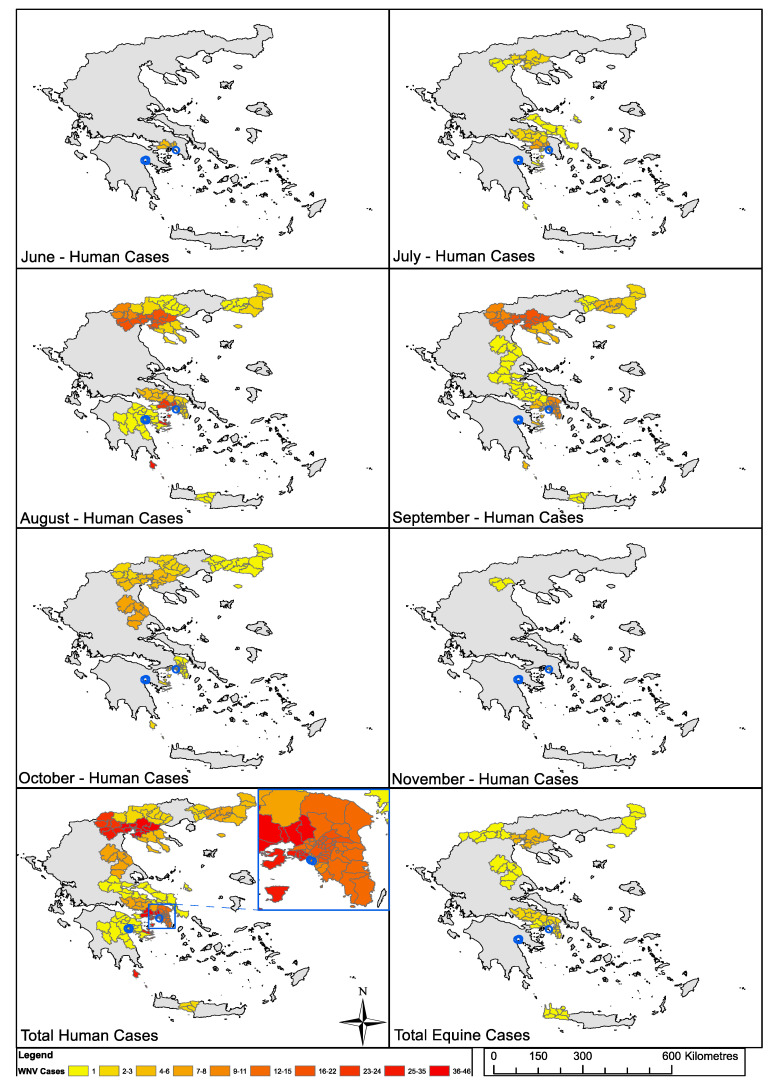
Reported human and equine cases of West Nile virus (WNV) in the 2018 transmission season. Maps were constructed in ArcMap 10.5 (ArcGIS, Esri, Redlands, USA) using Global Administrative layers for Greece (level 3), downloaded from www.gadm.org (Version 3.6) and ECDC WNV case report data from “Transmission of West Nile virus, June to December 2018—Table of cases, 2018 transmission season” downloaded from www.ecdc.europa.eu. The data from the ECDC surveillance Atlas was collected for each week of the transmission season, for human and equine cases, and then combined for each region, to generate monthly maps of human case reports, and cumulative total maps for human and equine cases. Mosquito sampling locations shown as blue circles, with collection locations in the Regional Unit of Argolida in the Peloponnese region to the west, and those in the Regional Unit of Palaio Faliro in the Attica region to the east.

The reported human cases of WNV during the 2018 transmission season revealed only two human cases in the Peloponnese region all year, and specifically in the area of Argolida just one human case was recorded which occurred in August (Figure 3). In the Attica region, however, a total of 159 human cases and 4 equine outbreaks were recorded during the transmission season, with the first reported human cases occurring in June. Specifically, there were 11 human cases reported in the municipality unit in which Palaio Faliro is located, occurring between August and November.

## 4. Discussion

Our mosquito trapping experiments using different adult traps show that in both regions BG traps collected both a larger number of mosquitoes of all species, and a greater number of individuals from the *Cx. pipiens* complex (although this was not statistically significant in the RU of Palaio Faliro). Previous trap comparison studies undertaken in Europe report contrasting results, ranging from BG traps in Germany collecting more *Cx. pipiens* complex mosquitoes than CDC and EVS traps [53], to a study in Spain showing no statistically significant differences between BG and CDC traps in collecting specimens from this complex [54]. CDC light traps are the most commonly used method for the surveillance of mosquito populations and are effective at trapping night-biting species from the *Culex* and *Anopheles* genera. In contrast, BG and EVS traps can be more effective against day-biting mosquitoes (using CO2 instead of a light source for attraction), including *Ae. albopictus* [55]. In addition, BG traps can be used with chemical lures that increase their trapping efficiency by imitating the olfactory cues of potential hosts. As expected, our results highlight that using a variety of trapping types can increase the species diversity of collections. However, a greater number of target vector species, such as individuals of the *Cx. pipiens* complex and invasive *Aedes* species (e.g., *Ae. albopictus*) could potentially be targeted using BG traps when capacity and resources are limited.

Although different mosquito species (across multiple genera) have been demonstrated to be competent vectors of WNV [6], the major vectors for WNV belong to the *Cx*. *pipiens* complex. In this study, we collected individuals of the *Cx. pipiens* complex in addition to other species including *Ae. albopictus*, *Cs. longiareolata* and *Ae. caspius* shown previously to be present in Greece [56,57,58]. The presence of the *pipiens* biotype, *molestus* biotype and hybrids in both the Attica and Peloponnese regions is consistent with previous studies in Greece [22,49,50]. We found variation in the prevalence of the different biotypes with the *pipiens* biotype comprising 54.5% (*n* = 79) in the RU of Palaio Faliro, 20.0% (*n* = 20) *molestus* biotype and 25.5% (*n* = 37) of hybrids. These results differ from another study that had found a more homogeneous *molestus* biotype population [44] which could be due to seasonality of collections as this study collected later into the transmission season (August–September 2010). In the RU of Argolida the biotypes of the *Cx. pipiens* complex were 68.1% (*n* = 98) of *pipiens* biotype, 8.3% (*n* = 12) of *molestus* biotype and 23.6% (*n* = 34) of *molestus* and *pipiens* hybrids. The high percentage of hybrids in this RU is similar to a previous study conducted in the area after the 2017 outbreak which reported 37% hybrids, 41% *pipiens* and 22% *molestus* biotypes [59].

In the USA, the high number of WNV cases in humans was correlated to the high number of hybrids [60]. Europe is considered to have more “pure” types but hybridization can result in a catholic feeding behaviour (feeding both on birds and mammals) increasing the risk of mixed populations acting as bridge-vectors of WNV between birds and humans/equines [44]. The feeding patterns of the different mosquito species, and the different biotypes within the species complex, are important in order to identify the contribution of each vector to both the enzootic maintenance of WNV in avian hosts, and the spill-over transmission to humans and horses [61]. In northern Greece, the predominance of the *pipiens* biotype could be facilitating the maintenance of the enzootic cycle of the virus between mosquitoes and birds in the area [44]. The presence of the *molestus* biotype and the existence of hybrids can promote an opportunistic biting behaviour that could contribute to the spill-over of infection to humans and equines.

In our study, we also collected several other species that have been implicated or shown to be potential WNV vectors. Experimental transmission has been shown for both *Cs. longiareolata* and *Ae. albopictus* [1] whereas laboratory experiments indicated that *Ae. caspius* may be incapable of transmitting WNV [33,62]. However, in some countries the high densities and detection of WNV in wild-caught specimens, have suggested this species may have a potential role in transmission, particularly during an outbreak when the level of viral circulation is high [63]. The presence of *Ae. albopictus*, an invasive species that has expanded its range across Europe since the late 1970s, would suggest the potential for transmission of additional arboviruses. *Aedes albopictus* has the ability to adapt to colder temperatures and stay dormant during the winter, and has previously been shown to be responsible for chikungunya virus outbreaks in Italy in 2007 [64]. In Greece, since its first reported presence in 2003 in the western part of the country, this species has now spread to almost every district [57]. *Aedes albopictus* has also been the principle vector responsible for dengue virus outbreaks in Hawaii in 2001–2002 and Mauritius in 2009 [65,66] and is a potential vector of Zika virus [67,68]. Dengue virus was detected in *Ae. albopictus* in Spain in 2015 [69] highlighting the potential for this species to contribute to transmission in Europe. Furthermore, it can be a competent vector of WNV when experimentally tested in laboratory conditions [70] although it has never been recorded as a WNV vector in the field, possibly due to its low propensity to bite birds [58].

Detection of WNV cDNA in four unfed *Cx. pipiens* complex specimens would indicate circulation of WNV in the RU of Argolida during our collection period in May. This represents the minimum number of positive individuals given the possibility of low virus levels being beyond the sensitivity (detection limit) of this PCR assay. This is interesting when compared to the spatial and temporal records of human and equine cases during 2018 (Figure 3) as only one human case was recorded from this area of the Peloponnese region all year, and not until August, suggesting WNV may have been circulating in the area for months before resulting in a case of human clinical disease. Interestingly, no equine cases were reported in this area for the 2018 transmission season despite WNV-infected mosquitoes collected from private houses in Dalamanara in close proximity to a third site containing horses (Table S1). The level of urbanization is likely a factor given the *molestus* biotype is considered more anthropophilic and present in urban areas compared to the more ornithophilic *pipiens* biotype more often found in rural areas. The confirmation that three of the positives were *pipiens* biotype, supports the possibility of virus circulating in an enzootic cycle, between birds and mosquitoes. However, the presence of WNV in one of the hybrids also demonstrates the potential for spill-over transmission to humans and equines in the area at this early time in the season. In comparison, no WNV was detected in mosquitoes collected from the RU of Palaio Faliro, but this area subsequently recorded a far greater number of human and equine cases during 2018. Across the whole Attica region, a total of 159 human cases were recorded, with the first reported cases occurring in June, and in the area in which Palaio Faliro is located, 11 human cases were reported, occurring between August and November. This highlights the likely variations in spatial and temporal transmission dynamics between these two very different localities, and the variable factors that can influence risk of host infection and subsequent disease during the transmission season.

## 5. Conclusions

Sampling during the onset of the 2018 WNV at pre-disease stage in the RUs in the Attica and Peloponnese regions was particularly important in a year in which more than 300 human cases were recorded in Greece. These results, combined with previous entomological surveys conducted in Greece, show the high occurrence of hybrids between the *pipiens* and *molestus* biotypes of *Cx. pipiens.* Previous studies have demonstrated the importance of hybrids as bridge vectors of WNV. Their role in spill-over transmission to humans, and the presence of hybrids (and WNV infections) in RUs in the Attica and Peloponnese regions of Greece suggest these areas are vulnerable to outbreaks. Furthermore, 2018 was the first year in Greece in which WNV human cases were recorded so early in the transmission period with six human cases confirmed by late June. Future entomological surveillance studies should incorporate molecular xenomonitoring to determine this potential expansion of the transmission season to provide early warning systems for potential WNV outbreaks. Notification of human WNV cases in Europe through The European Surveillance System (TESSy) [71] of the ECDC allows weekly mapping of human cases [27]. In addition, reporting of WNV encephalomyelitis in horses to the European Commission is carried out via the Animal Disease Notification System (ADNS). As reported cases of WNV infection in humans have been from southern and central European countries and a majority of human infections are asymptomatic, it is particularly important to undertake entomological and avian surveillance to determine if WNV circulation is occurring in a particular area, as a precursor to potential spill-over transmission to humans and other mammals. In particular, entomological surveys to determine the distribution of mosquito vectors such as *Cx. pipiens* through the Pan-European VectorNet [72] will play a crucial role in an integrated approach to WNV surveillance and control efforts to minimise the impact of outbreaks on veterinary and public health.

## Figures and Tables

**Table 1:** Total mosquitoes collected from different locations in the Attica and Peloponnese regions of Greece using Biogents Sentinel (BG) traps, Encephalitis Vector Survey (EVS) traps and CDC traps. Mosquitoes were morphologically identified using keys and females were classified as non-blood-fed (no visible blood in abdomen), blood-fed or gravid.

Region/Regional Unit	Sampling Location	Species/Complex	Mosquitoes Collected					
			Females			Males	Total	% of Total Per Site
			Non-Blood-Fed	Blood-Fed	Gravid			
Attica/Palaio Faliro	Rema Pikrodafnis	*Cx. pipiens* complex	68	6	3	1	78	55.7
		*Ae. albopictus*	17	0	0	33	50	35.7
		*Cs. longiareolata*	1	1	0	7	9	6.4
		Unidentified	3	0	0	0	3	2.1
	Dimarchio	*Cx. pipiens* complex	47	1	0	0	48	64.9
		*Ae. albopictus*	8	0	0	5	13	17.6
		*Cs. longiareolata*	0	1	0	12	13	17.6
	KAPI	*Cx. pipiens* complex	106	2	2	8	118	84.3
		*Ae. albopictus*	4	1	0	9	14	10.0
		*Cs. longiareolata*	2	1	0	4	7	5.0
		Unidentified	1	0	0	0	1	0.7
Peloponnese/Argolida	Agia Triada	*Cx. pipiens* complex	101	2	3	9	115	54.0
		*Ae. albopictus*	1	0	0	0	1	0.5
		*Cs. longiareolata*	31	0	0	64	95	44.6
		*Ae. caspius*	1	0	0	0	1	0.5
		*An. maculipennis* complex	0	0	0	1	1	0.5
	Nea Tirintha	*Cx. pipiens* complex	140	3	0	4	147	49.0
		*Cs. longiareolata*	14	1	0	23	38	12.7
		*Ae. caspius*	91	2	0	1	94	31.3
		*An. maculipennis* complex	13	3	0	0	16	5.3
		Unidentified	5	0	0	0	5	1.7
	Dalamanara	*Cx. pipiens* complex	153	4	1	0	158	81.0
		*Ae. albopictus*	1	0	0	0	1	0.5
		*Cs. longiareolata*	5	0	0	4	9	4.6
		*Ae. caspius*	22	0	0	0	22	11.3
		*An. maculipennis* complex	2	0	0	0	2	1.0
		Unidentified	3	0	0	0	3	1.5
Total collected			840	28	9	185	1062	–

**Table 2:** Mann–Whitney statistical analysis comparing the number of *Cx. pipiens* complex mosquitoes collected using three traps.

Region/Regional Unit	Trap Comparison ^1^	U-Value	Z-Score	*p*-Value
Attica/Palaio Faliro	BG vs. CDC	258.0	1.834	0.07
	BG vs EVS	218.5	2.517	0.01
	CDC vs. EVS	342.5	0.372	0.71
Peloponnese/ Argolida	BG vs. CDC	40.0	2.256	0.02
	BG vs EVS	32.0	2.667	0.01
	CDC vs. EVS	76.5	0.385	0.70

^1^ Biogents Sentinel traps (BG traps), Centre for Disease Control miniature light traps (CDC traps) and Heavy-Duty Encephalitis Vector Survey traps (EVS traps).

**Table 3:** *CO1* GenBank accession numbers for representatives of species confirmed by molecular identification. The location, species and *CO1* gene fragment in addition to the accession number on GenBank are shown.

Specimen Code	Sampling Location	Morphological Identification	*CO1* Gene Fragment (Reference)	GenBank Accession Number
AT1	Agia Triada	*Cx. pipiens*	[49]	MN005042
RP1	Rema Pikrodafnis	*Cx. pipiens*	[49]	MN005043
DI1	Dimarchio	*Cx. pipiens*	[49]	MN005044
DA1	Dalamanara	*Cx. pipiens*	[49]	MN005045
KA1	Kapi	*Cx. pipiens*	[49]	MN005046
NT1	Nea Tirintha	*Cx. pipiens*	[49]	MN005047
RP2	Rema Pikrodafnis	*Cs. longiareolata*	[48]	MN005048
DA2	Dalamanara	*Cs. longiareolata*	[48]	MN005049
AT2	Agia Triada	*Cs. longiareolata*	[48]	MN005050
NT2	Nea Tirintha	*Ae. caspius*	[47]	MN005051
AT3	Agia Triada	*Ae. caspius*	[47]	MN005052
DA3	Dalamanara	*Ae. caspius*	[47]	MN005053
DI2	Dimarchio	*Ae. albopictus*	[48]	MN005054
AT4	Agia Triada	*Ae. albopictus*	[48]	MN005055
RP3	Rema Pikrodafnis	*Ae. albopictus*	[48]	MN005056

## Supplementary Materials

The following are available online at https://www.mdpi.com/2075-4450/11/6/329/s1, Table S1: Geographical locations with GPS co-ordinates of mosquito trapping sites within the Attica and Peloponnese regions of Greece.

## References

[1] Hubalek Z., Halouzka J. (1999). West Nile fever—A reemerging mosquito-borne viral disease in Europe. Emerg. Infect. Dis..

[2] Hubalek Z. (2008). Mosquito-borne viruses in Europe. Parasitol. Res..

[3] Brustolin M., Talavera S., Santamaria C., Rivas R., Pujol N., Aranda C., Marques E., Valle M., Verdun M., Pages N. (2016). Culex pipiens and Stegomyia albopicta (=Aedes albopictus) populations as vectors for lineage 1 and 2 West Nile virus in Europe. Med. Vet. Entomol..

[4] Hayes E.B., Komar N., Nasci R.S., Montgomery S.P., O’Leary D.R., Campbell G.L. (2005). Epidemiology and transmission dynamics of West Nile virus disease. Emerg. Infect. Dis..

[5] Vilibic-Cavlek T., Savic V., Petrovic T., Toplak I., Barbic L., Petric D., Tabain I., Hrnjakovic-Cvjetkovic I., Bogdanic M., Klobucar A. (2019). Emerging trends in the epidemiology of West Nile and Usutu virus infections in Southern Europe. Front. Vet. Sci..

[6] Ciota A.T. (2017). West Nile virus and its vectors. Curr. Opin. Insect. Sci..

[7] Kramer L.D., Ciota A.T., Kilpatrick A.M. (2019). Introduction, spread, and establishment of West Nile virus in the Americas. J. Med. Entomol..

[8] Vogels C.B.F., Hartemink N., Koenraadt C.J.M. (2017). Modelling West Nile virus transmission risk in Europe: Effect of temperature and mosquito biotypes on the basic reproduction number. Sci. Rep..

[9] Hayes E.B., Sejvar J.J., Zaki S.R., Lanciotti R.S., Bode A.V., Campbell G.L. (2005). Virology, pathology, and clinical manifestations of West Nile virus disease. Emerg. Infect. Dis..

[10] Custer B., Busch M.P., Marfin A.A., Petersen L.R. (2005). The cost-effectiveness of screening the U.S. blood supply for West Nile virus. Ann. Intern. Med..

[11] Korves C.T., Goldie S.J., Murray M.B. (2006). Cost-effectiveness of alternative blood-screening strategies for West Nile virus in the United States. PLoS Med..

[12] Bowen R.A., Bosco-Lauth A., Syvrud K., Thomas A., Meinert T.R., Ludlow D.R., Cook C., Salt J., Ons E. (2014). Protection of horses from West Nile virus Lineage 2 challenge following immunization with a whole, inactivated WNV lineage 1 vaccine. Vaccine.

[13] Long M.T., Gibbs E.P., Mellencamp M.W., Bowen R.A., Seino K.K., Zhang S., Beachboard S.E., Humphrey P.P. (2007). Efficacy, duration, and onset of immunogenicity of a West Nile virus vaccine, live Flavivirus chimera, in horses with a clinical disease challenge model. Equine. Vet. J..

[14] Smithburn K.C., Hughes T.P., Burke A.W., Paul J.H. (1940). A neurotropic virus isolated from the blood of a native of Uganda1. Am. J. Trop. Med. Hyg..

[15] Gubler D.J. (2007). the continuing spread of West Nile virus in the Western Hemisphere. Clin. Infect. Dis..

[16] Grubaugh N.D., Ebel G.D. (2016). Dynamics of West Nile virus evolution in mosquito vectors. Curr. Opin. Virol..

[17] Colpitts T.M., Conway M.J., Montgomery R.R., Fikrig E. (2012). West Nile virus: Biology, transmission, and human infection. Clin. Microbiol. Rev..

[18] Petersen L.R., Marfin A.A. (2002). West Nile virus: A primer for the clinician. Ann. Intern. Med..

[19] Tantely M.L., Goodman S.M., Rakotondranaivo T., Boyer S. (2016). Review of West Nile virus circulation and outbreak risk in Madagascar: Entomological and ornithological perspectives. Parasite.

[20] Levine R.S., Mead D.G., Kitron U.D. (2013). Limited spillover to humans from West Nile Virus viremic birds in Atlanta, Georgia. Vector Borne Zoonotic Dis..

[21] Jourdain E., Schuffenecker I., Korimbocus J., Reynard S., Murri S., Kayser Y., Gauthier-Clerc M., Sabatier P., Zeller H.G. (2007). West Nile virus in wild resident birds, Southern France, 2004. Vector Borne Zoonotic Dis..

[22] Linke S., Niedrig M., Kaiser A., Ellerbrok H., Muller K., Muller T., Conraths F.J., Muhle R.U., Schmidt D., Koppen U. (2007). Serologic evidence of West Nile virus infections in wild birds captured in Germany. Am. J. Trop. Med. Hyg..

[23] Figuerola J., Soriguer R., Rojo G., Gomez Tejedor C., Jimenez-Clavero M.A. (2007). Seroconversion in wild birds and local circulation of West Nile virus, Spain. Emerg. Infect. Dis..

[24] Calzolari M., Gaibani P., Bellini R., Defilippo F., Pierro A., Albieri A., Maioli G., Luppi A., Rossini G., Balzani A. (2012). Mosquito, bird and human surveillance of West Nile and Usutu viruses in Emilia-Romagna Region (Italy) in 2010. PLoS ONE.

[25] Sambri V., Capobianchi M.R., Cavrini F., Charrel R., Donoso-Mantke O., Escadafal C., Franco L., Gaibani P., Gould E.A., Niedrig M. (2013). Diagnosis of West Nile virus human infections: Overview and proposal of diagnostic protocols considering the results of external quality assessment studies. Viruses.

[26] Tsai T.F., Popovici F., Cernescu C., Campbell G.L., Nedelcu N.I. (1998). West Nile encephalitis epidemic in southeastern Romania. Lancet.

[27] European Centre for Disease Prevention and Control Weekly Updates: 2018 West Nile Fever Transmission Season. https://ecdc.europa.eu/en/west-nile-fever/surveillance-and-disease-data/disease-data-ecdc.

[28] Papa A. (2013). West Nile virus infections in humans—Focus on Greece. J. Clin. Virol..

[29] Papa A., Perperidou P., Tzouli A., Castilletti C. (2010). West Nile virus—Neutralizing antibodies in humans in Greece. Vector Borne Zoonotic Dis..

[30] Danis K., Papa A., Theocharopoulos G., Dougas G., Athanasiou M., Detsis M., Baka A., Lytras T., Mellou K., Bonovas S. (2011). Outbreak of West Nile virus infection in Greece, 2010. Emerg. Infect. Dis..

[31] Hellenic Centre for Disease Control and Prevention (KEELPNO). https://ecdc.europa.eu/en/hellenic-centre-disease-control-and-prevention-keelpno-epiet.

[32] (2018). Epidemiological Update: West Nile Virus Transmission Season in Europe. https://ecdc.europa.eu/en/news-events/epidemiological-update-west-nile-virus-transmission-season-europe-2018.

[33] Vogels C.B., Goertz G.P., Pijlman G.P., Koenraadt C.J. (2017). Vector competence of European mosquitoes for West Nile virus. Emerg. Microbes Infect..

[34] Jansen S., Heitmann A., Luhken R., Leggewie M., Helms M., Badusche M., Rossini G., Schmidt-Chanasit J., Tannich E. (2019). Culex torrentium: A Potent vector for the transmission of West Nile Virus in Central Europe. Viruses.

[35] Schaffner F., Versteirt V., Medlock J. (2014). Guidelines for the Surveillance of Native Mosquitoes in Europe.

[36] Anderson J.F., Main A.J., Ferrandino F.J. (2020). Horizontal and Vertical Transmission of West Nile Virus by Aedes vexans (Diptera: Culicidae). J. Med. Entomol..

[37] Smith J.L., Fonseca D.M. (2004). Rapid assays for identification of members of the Culex (Culex) pipiens complex, their hybrids, and other sibling species (Diptera: *Culicidae*). Am. J. Trop. Med. Hyg..

[38] Becker N., Jost A., Weitzel T. (2012). The Culex pipiens complex in Europe. J. Am. Mosq. Control. Assoc..

[39] Hesson J.C., Rettich F., Merdic E., Vignjevic G., Ostman O., Schafer M., Schaffner F., Foussadier R., Besnard G., Medlock J. (2014). The arbovirus vector Culex torrentium is more prevalent than Culex pipiens in northern and central Europe. Med. Vet. Entomol..

[40] Kent R.J., Harrington L.C., Norris D.E. (2007). Genetic differences between Culex pipiens f. molestus and Culex pipiens pipiens (Diptera: Culicidae) in New York. J. Med. Entomol..

[41] Borstler J., Jost H., Garms R., Kruger A., Tannich E., Becker N., Schmidt-Chanasit J., Luhken R. (2016). Host-feeding patterns of mosquito species in Germany. Parasit Vectors.

[42] Fritz M.L., Walker E.D., Miller J.R., Severson D.W., Dworkin I. (2015). Divergent host preferences of above- and below-ground Culex pipiens mosquitoes and their hybrid offspring. Med. Vet. Entomol..

[43] Osorio H.C., Ze-Ze L., Alves M.J. (2012). Host-feeding patterns of Culex pipiens and other potential mosquito vectors (Diptera: Culicidae) of West Nile virus (Flaviviridae) collected in Portugal. J. Med. Entomol..

[44] Gomes B., Kioulos E., Papa A., Almeida A.P., Vontas J., Pinto J. (2013). Distribution and hybridization of Culex pipiens forms in Greece during the West Nile virus outbreak of 2010. Infect. Genet. Evol..

[45] Shaikevich E.V., Vinogradova E.B. (2014). The discovery of a hybrid population of mosquitoes of the *Culex pipiens* L. complex (Diptera, Culicidae) on the Kos Island (Greece) by means of molecular markers. Entomol. Rev..

[46] Samanidou-Voyadjoglou A., Harbach R.E. (2001). Keys to the adult female mosquitoes (Culicidae) of Greece. EMB.

[47] Kumar N.P., Rajavel A.R., Natarajan R., Jambulingam P. (2007). DNA barcodes can distinguish species of Indian mosquitoes (Diptera: Culicidae). J. Med. Entomol..

[48] Folmer O., Black M., Hoeh W., Lutz R., Vrijenhoek R. (1994). DNA primers for amplification of mitochondrial cytochrome c oxidase subunit I from diverse metazoan invertebrates. Mol. Mar. Biol. Biotechnol..

[49] Zittra C., Flechl E., Kothmayer M., Vitecek S., Rossiter H., Zechmeister T., Fuehrer H.P. (2016). Ecological characterization and molecular differentiation of Culex pipiens complex taxa and Culex torrentium in eastern Austria. Parasit Vectors.

[50] Bahnck C.M., Fonseca D.M. (2006). Rapid assay to identify the two genetic forms of *Culex (Culex) pipiens* L. (Diptera: Culicidae) and hybrid populations. Am. J. Trop. Med. Hyg..

[51] Kumar S., Stecher G., Tamura K. (2016). MEGA7: Molecular Evolutionary Genetics Analysis Version 7.0 for bigger datasets. Mol. Biol. Evol..

[52] Linke S., Ellerbrok H., Niedrig M., Nitsche A., Pauli G. (2007). Detection of West Nile virus lineages 1 and 2 by real-time PCR. J. Virol. Methods.

[53] Luhken R., Pfitzner W.P., Borstler J., Garms R., Huber K., Schork N., Steinke S., Kiel E., Becker N., Tannich E. (2014). Field evaluation of four widely used mosquito traps in Central Europe. Parasit Vectors.

[54] Roiz D., Roussel M., Munoz J., Ruiz S., Soriguer R., Figuerola J. (2012). Efficacy of mosquito traps for collecting potential West Nile mosquito vectors in a natural Mediterranean wetland. Am. J. Trop. Med. Hyg..

[55] Meeraus W.H., Armistead J.S., Arias J.R. (2008). Field comparison of novel and gold standard traps for collecting Aedes albopictus in Northern Virginia. J. Am. Mosq. Control. Assoc..

[56] Darsie R.F., Samanidou-Voyadjoglou A. (1997). Keys for the identification of the mosquitoes of Greece. J. Am. Mosq. Control Assoc..

[57] Badieritakis E., Papachristos D., Latinopoulos D., Stefopoulou A., Kolimenakis A., Bithas K., Patsoula E., Beleri S., Maselou D., Balatsos G. (2018). Aedes albopictus (Skuse, 1895) (Diptera: Culicidae) in Greece: 13 years of living with the Asian tiger mosquito. Parasitol. Res..

[58] Kioulos I., Michaelakis A., Kioulos N., Samanidou-Voyadjoglou A., Koliopoulos G. (2014). Mosquito (Diptera: Culicidae) fauna in natural breeding sites of Attica basin, Greece. Hell. Plant Prot. J..

[59] Mavridis K., Fotakis E.A., Kioulos I., Mpellou S., Konstantas S., Varela E., Gewehr S., Diamantopoulos V., Vontas J. (2018). Detection of West Nile Virus—Lineage 2 in Culex pipiens mosquitoes, associated with disease outbreak in Greece, 2017. Acta Trop..

[60] Ciota A.T., Chin P.A., Kramer L.D. (2013). The effect of hybridization of Culex pipiens complex mosquitoes on transmission of West Nile virus. Parasit Vectors.

[61] Molaei G., Andreadis T.G., Armstrong P.M., Anderson J.F., Vossbrinck C.R. (2006). Host feeding patterns of Culex mosquitoes and West Nile virus transmission, northeastern United States. Emerg. Infect. Dis..

[62] Balenghien T., Vazeille M., Grandadam M., Schaffner F., Zeller H., Reiter P., Sabatier P., Fouque F., Bicout D.J. (2008). Vector competence of some French Culex and Aedes mosquitoes for West Nile virus. Vector Borne Zoonotic Dis..

[63] Mancini G., Montarsi F., Calzolari M., Capelli G., Dottori M., Ravagnan S., Lelli D., Chiari M., Santilli A., Quaglia M. (2017). Mosquito species involved in the circulation of West Nile and Usutu viruses in Italy. Vet. Ital..

[64] Paupy C., Delatte H., Bagny L., Corbel V., Fontenille D. (2009). Aedes albopictus, an arbovirus vector: From the darkness to the light. Microbes Infect..

[65] Effler P.V., Pang L., Kitsutani P., Vorndam V., Nakata M., Ayers T., Elm J., Tom T., Reiter P., Rigau-Perez J.G. (2005). Dengue fever, Hawaii, 2001–2002. Emerg. Infect. Dis..

[66] Ramchurn S.K., Moheeput K., Goorah S.S. (2009). An analysis of a short-lived outbreak of dengue fever in Mauritius. Euro Surveill..

[67] Wong P.S., Li M.Z., Chong C.S., Ng L.C., Tan C.H. (2013). Aedes (Stegomyia) albopictus (Skuse): A potential vector of Zika virus in Singapore. PLoS Negl. Trop. Dis..

[68] Grard G., Caron M., Mombo I.M., Nkoghe D., Mboui Ondo S., Jiolle D., Fontenille D., Paupy C., Leroy E.M. (2014). Zika virus in Gabon (Central Africa)--2007: A new threat from Aedes albopictus?. PLoS Negl. Trop. Dis..

[69] Aranda C., Martinez M.J., Montalvo T., Eritja R., Navero-Castillejos J., Herreros E., Marques E., Escosa R., Corbella I., Bigas E. (2018). Arbovirus surveillance: First dengue virus detection in local Aedes albopictus mosquitoes in Europe, Catalonia, Spain, 2015. Euro Surveill..

[70] Fortuna C., Remoli M.E., Severini F., Di Luca M., Toma L., Fois F., Bucci P., Boccolini D., Romi R., Ciufolini M.G. (2015). Evaluation of vector competence for West Nile virus in Italian Stegomyia albopicta (=Aedes albopictus) mosquitoes. Med. Vet. Entomol..

[71] European Centre for Disease Prevention and Control (ECDC) The European Surveillance System (TESSy). https://www.ecdc.europa.eu/en/publications-data/european-surveillance-system-tessy.

[72] European Centre for Disease Prevention and Control European Network for Sharing Data on the Geographic Distribution of Arthropod Vectors, Transmitting Human and Animal Disease Agents (VectorNet). https://www.ecdc.europa.eu/en/about-us/partnerships-and-networks/disease-and-laboratory-networks/vector-net.

