# Supplementary material for: A Comparison of Adult Mosquito Trapping Methods to Assess Potential West Nile Virus Mosquito Vectors in Greece during the Onset of the 2018 Transmission Season
Source: Insects. 2020 May 27;11(6):329. doi: 10.3390/insects11060329 (PMC7348707; doi:10.3390/insects11060329)
Supplement: Supplementary file 1 [file insects-11-00329-s001.pdf]

Table S1. Geographical locations with GPS co-ordinates of mosquito trapping sites within the Attica and Peloponnese regions of Greece.

Region/Regional Unit	Sampling location (Local name/Description)	Trapping Site (Street Name or Description)	GPS Co-ordinates (Decimal Degrees)	
			Latitude	Longitude
Attica/Palaio Faliro	Rema Pikrodafnis	Aristeidou str.	37.923972	23.710106
		Dimokritou str.	37.923836	23.711511
		Sofokleous str.	37.922997	23.710306
	Dimarchio	Terpsichoris str.	37.928111	23.699008
		Naiadon str.	37.927989	23.696631
		Athanasiadou str.	37.928819	23.698006
	KAPI	Seirinon str.	37.931997	23.692625
		Esperou str.	37.931228	23.692983
		Atlantos str.	37.931408	23.692219
Peloponnese/ Argolida	Agia Triada	Veterinary clinic	37.636256	22.798003
		Juice factory	37.6439366	22.7883761
		Private house	37.638997	22.805275
	Nea Tirintha	Guard room (prisons)	37.596544	22.799989
		Sheep area (prisons)	37.594242	22.796617
		Cattle area (prisons)	37.592711	22.797669
	Dalamanara	Horse area	37.611461	22.739725
		Private house 1	37.620261	22.737842
		Private house 2	37.612106	22.738719
